# Supplementary material for: Reservoir displacement by an invasive rodent reduces Lassa virus zoonotic spillover risk
Source: Nat Commun. 2024 Apr 27;15:3589. doi: 10.1038/s41467-024-47991-1 (PMC11055883; doi:10.1038/s41467-024-47991-1)
Supplement: Supplementary file 1 — Supplementary Information [file 41467_2024_47991_MOESM1_ESM.pdf]

Supplementary Information
for
Reservoir displacement by an invasive rodent reduces Lassa virus zoonotic spillover risk

Evan A. Eskew[1], Brian H. Bird[2], Bruno M. Ghersi[2, 3], James Bangura[4], Andrew J. Basinski[1], Emmanuel Amara[4], Mohamed A. Bah[5], Marilyn C. Kanu[4], Osman T. Kanu[4], Edwin G. Lavalie[4], Victor Lungay[4], Willie Robert[4], Mohamed A. Vandi[6], Elisabeth Fichet-Calvet[7], and Scott L. Nuismer[8]

[1]Institute for Interdisciplinary Data Sciences, University of Idaho, Moscow, ID, USA
[2]One Health Institute, School of Veterinary Medicine, University of California - Davis, Davis, CA, USA
[3]Cummings School of Veterinary Medicine, Tufts University, North Grafton, MA, USA
[4]University of Makeni, Makeni, Sierra Leone
[5]Ministry of Agriculture and Forestry, Freetown, Sierra Leone
[6]Ministry of Health and Sanitation, Freetown, Sierra Leone
[7]Bernhard Nocht Institute for Tropical Medicine, Hamburg, Germany
[8]Department of Biological Sciences, University of Idaho, Moscow, ID, USA

April 2024

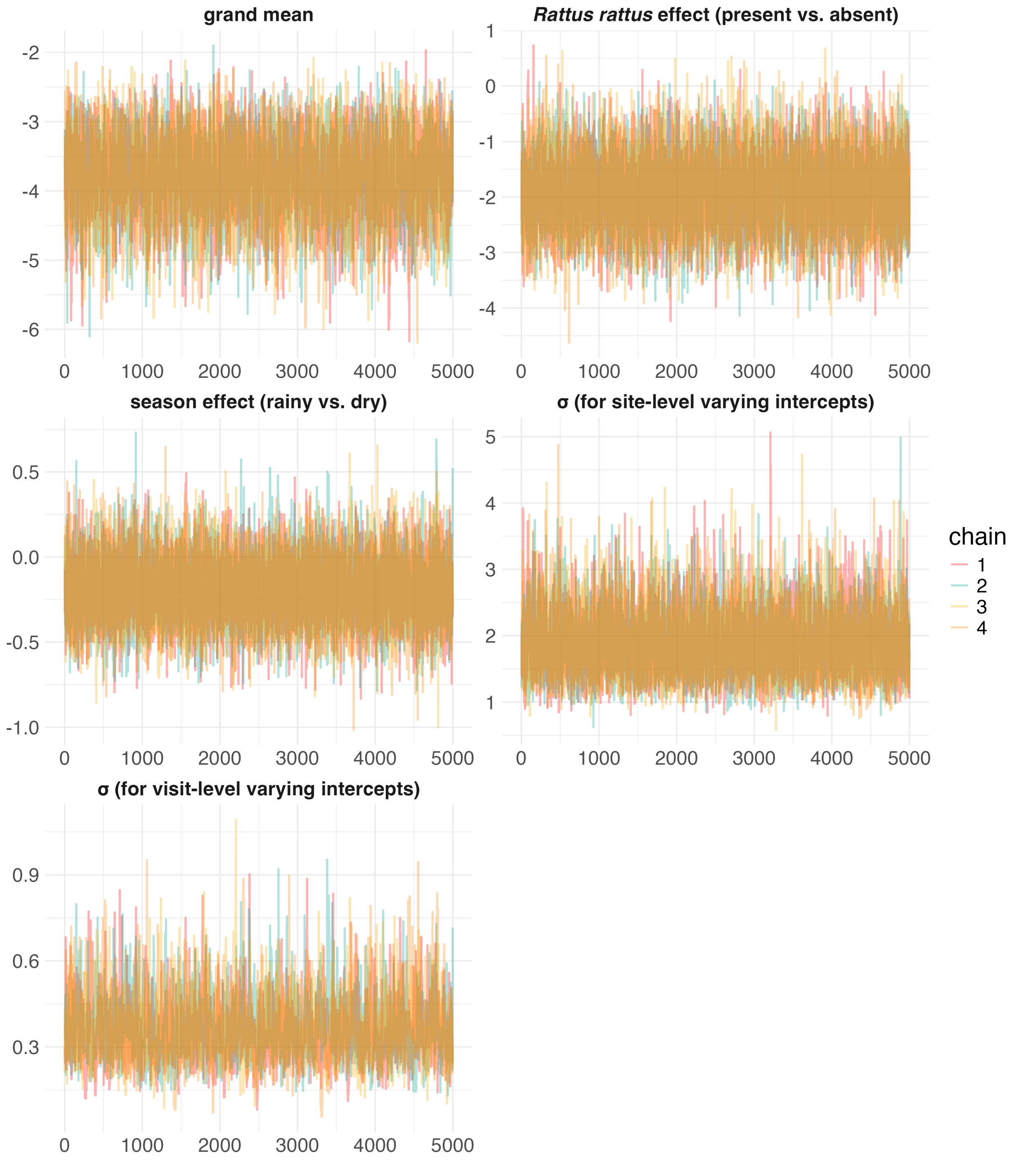

Figure S1: **Trace plots for select parameters from a visit-level Bayesian model of *Mastomys natalensis* catch using house trapping data.** Site- and visit-level varying intercept parameters are not displayed for clarity. Plots show traces across all four independent Markov chains, with each chain represented by a different color. Note that the y-axis scale differs across parameters.

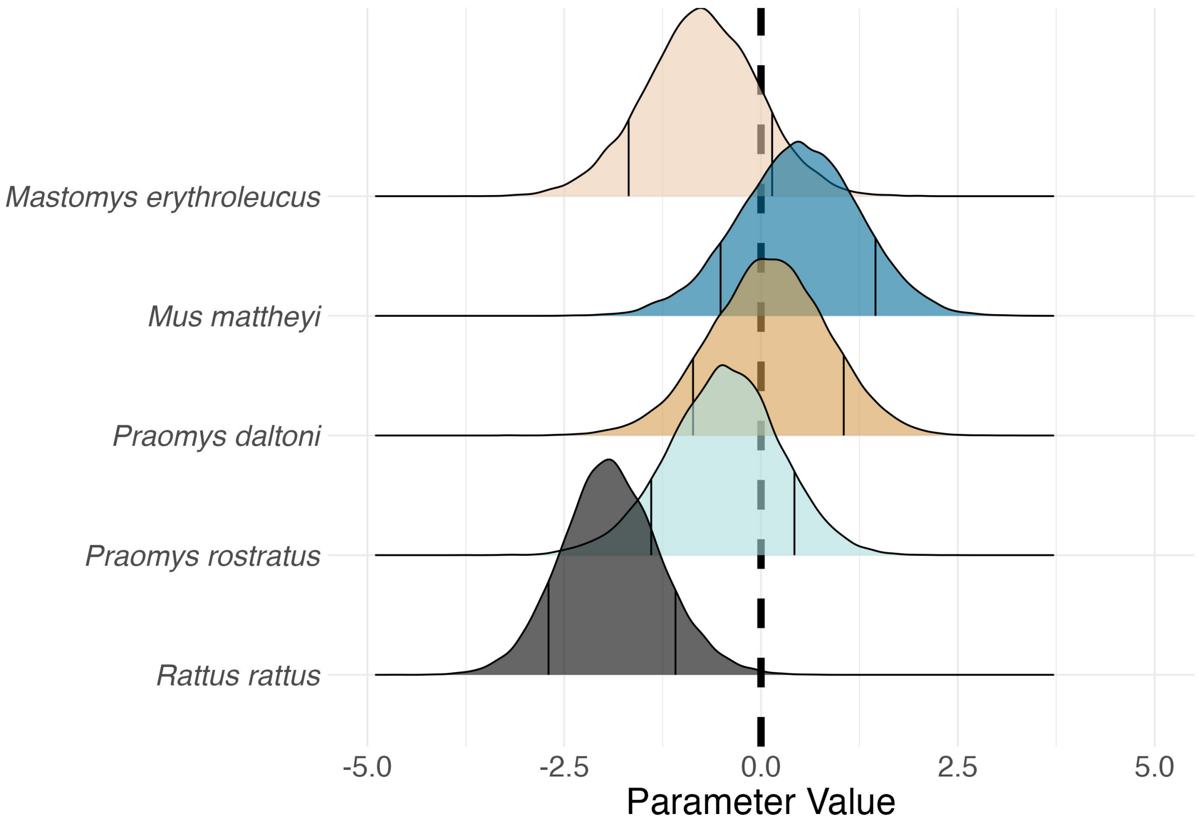

Figure S2: **Ridgeline plot showing estimated effects of the presence of five different rodent species on *Mastomys natalensis* catch using a visit-level Bayesian model and house trapping data.** To estimate these effects, we used a model identical to the visit-level Bayesian model described in the main text and Figure S11, swapping out alternative rodent species for the *R. rattus* presence effect (which is also shown here). All rodents with 100 or more captures in our full dataset were tested. The complete posterior distribution for each parameter is shown, and thin vertical lines within the distributions delineate the 80% percentile intervals (i.e., the 10th and 90th percentiles of each distribution).

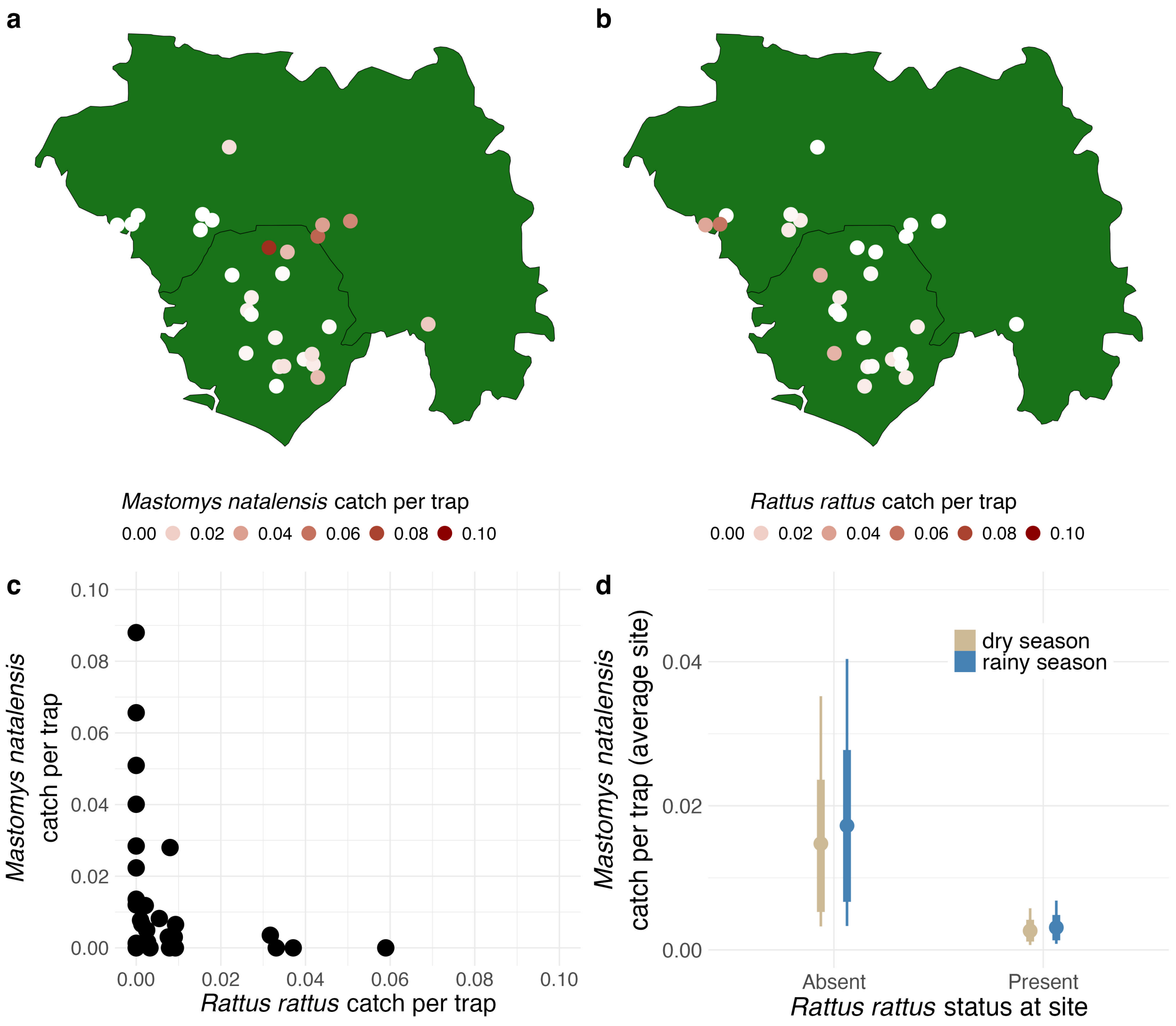

Figure S3: **Patterns of *Mastomys natalensis* and *Rattus rattus* catch per trap across 28 study sites in Sierra Leone and Guinea.** Map of catch per trap for *M. natalensis* (a) and *R. rattus* (b), and a scatterplot of the same data (c). Here, catch per trap was calculated using all trap-nights from a given site (i.e., inside and outside of houses; n = 36,759 trap-nights). Panel (d) shows the implied values of *M. natalensis* catch per trap for sites without and with *R. rattus* present, as derived from a visit-level Bayesian statistical model (n = 20,000 posterior samples; see main text for details). Colors indicate sampling season, points indicate posterior means, thick lines represent 90% HPDIs, and thin lines represent 99% HPDIs.

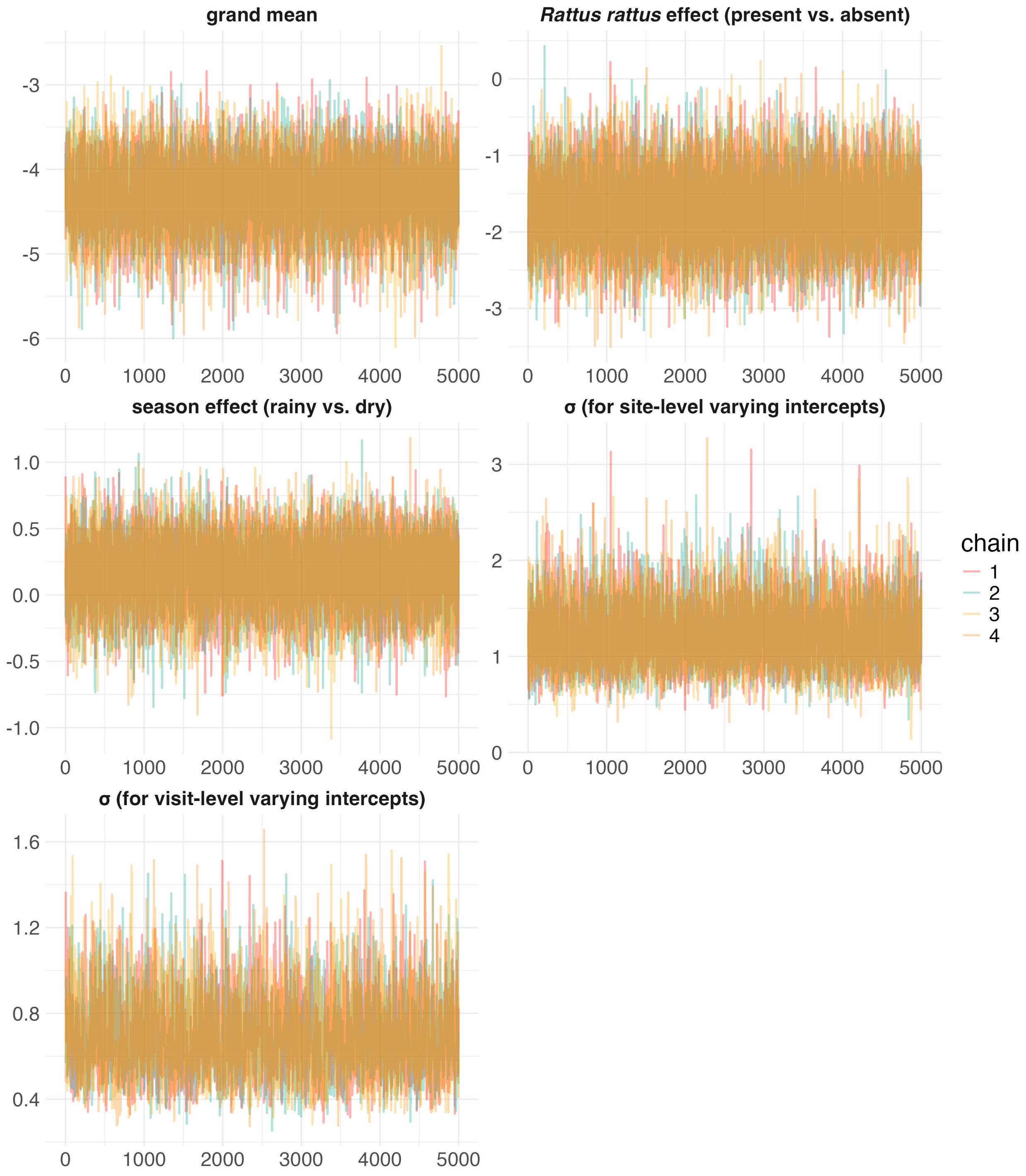

Figure S4: **Trace plots for select parameters from a visit-level Bayesian model of *Mastomys natalensis* catch using all trapping data.** Site- and visit-level varying intercept parameters are not displayed for clarity. Plots show traces across all four independent Markov chains, with each chain represented by a different color. Note that the y-axis scale differs across parameters.

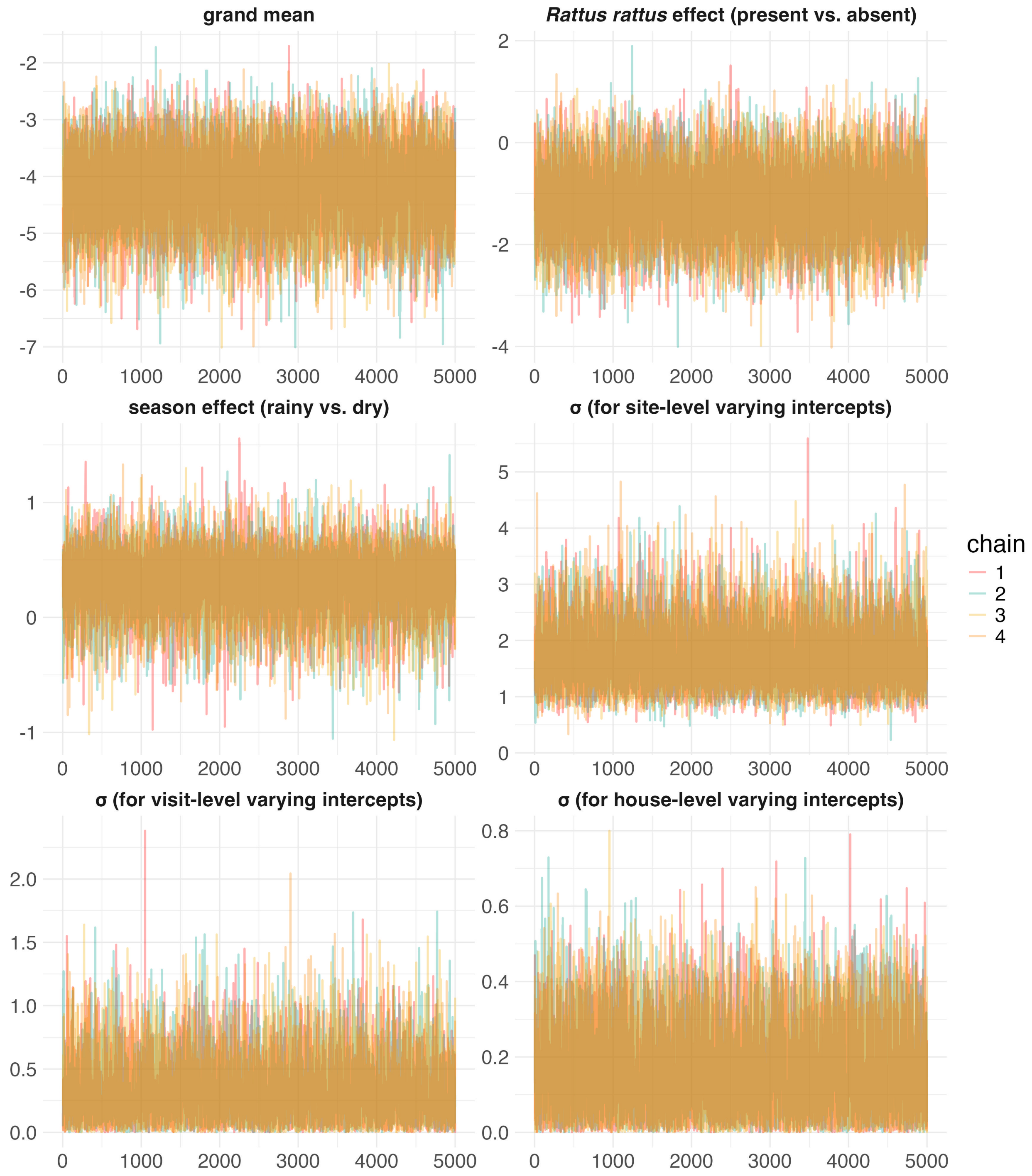

Figure S5: **Trace plots for select parameters from a house-level Bayesian model of *Mastomys natalensis* catch using only house trapping data from Sierra Leone and a site-level *Rattus rattus* predictor.** Site-, visit-, and house-level varying intercept parameters are not displayed for clarity. Plots show traces across all four independent Markov chains, with each chain represented by a different color. Note that the y-axis scale differs across parameters. In this model, the *R. rattus* effect is formulated as a site-level presence/absence variable (i.e., all houses at a site where *R. rattus* was detected are assumed to be exposed to *R. rattus*).

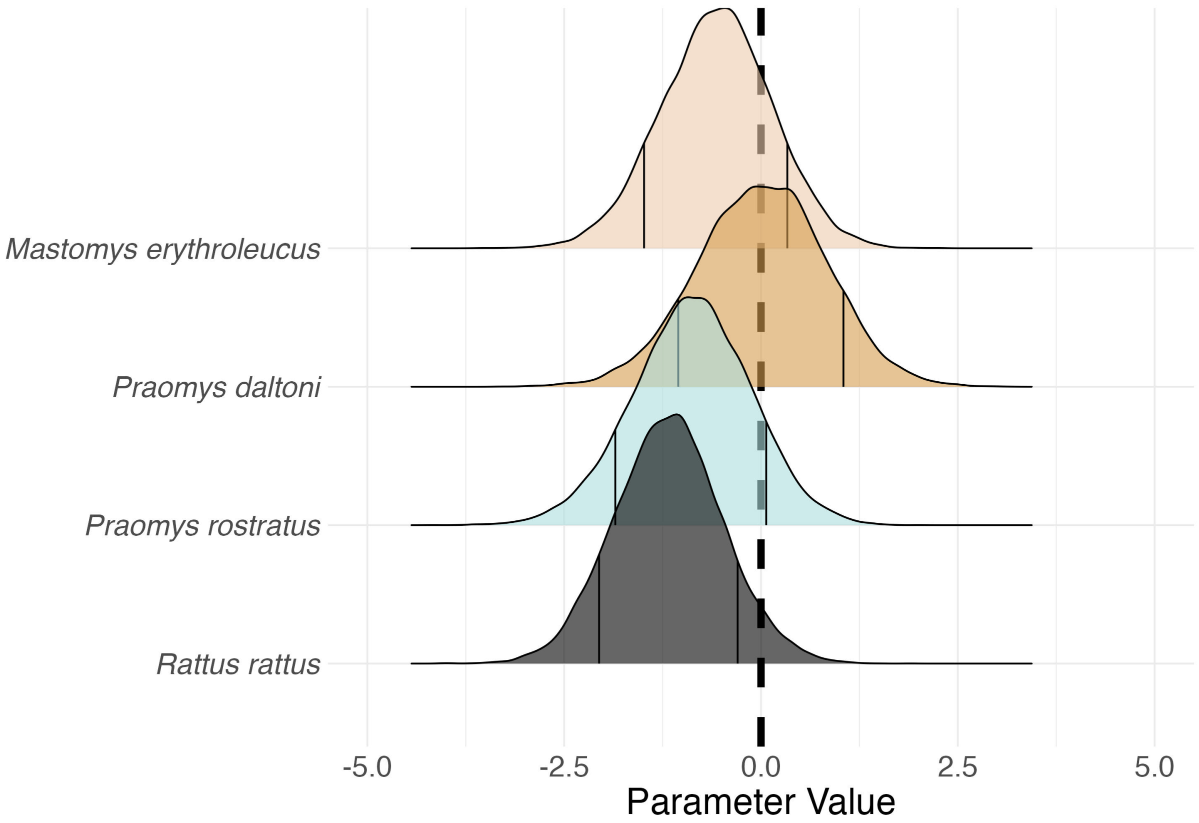

Figure S6: **Ridgeline plot showing estimated effects of the presence of four different rodent species on *Mastomys natalensis* catch using a house-level Bayesian model, house trapping data, and site-level rodent presence predictors.** To estimate these effects, we used a model identical to the house-level Bayesian model described in the main text, swapping out alternative rodent species for the *R. rattus* presence at site effect (which is also shown here). All rodents with 100 or more captures in our full dataset were tested, with the exception of *Mus mattheyi*, which was never captured in houses in Sierra Leone. The complete posterior distribution for each parameter is shown, and thin vertical lines within the distributions delineate the 80% percentile intervals (i.e., the 10th and 90th percentiles of each distribution).

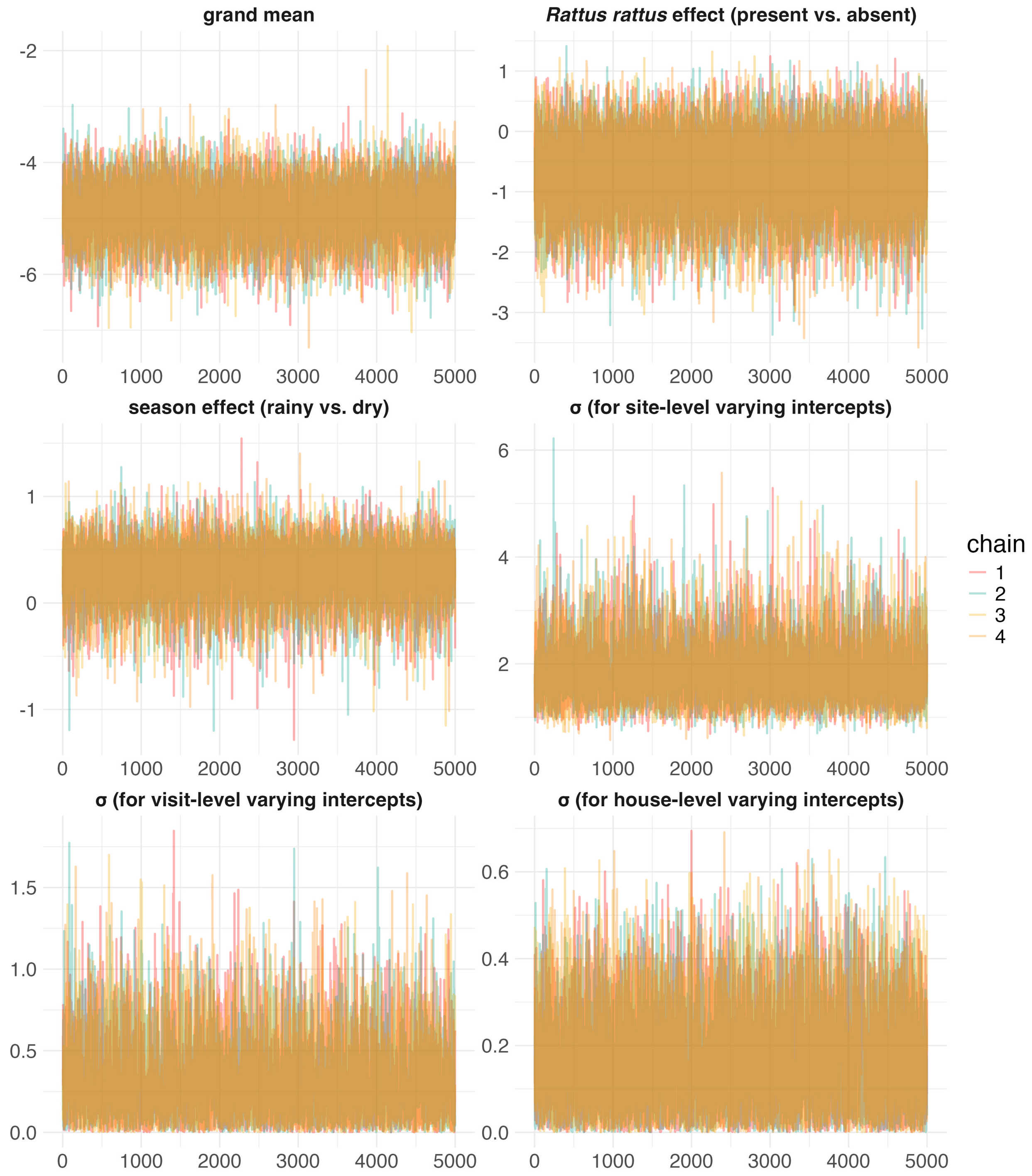

Figure S7: **Trace plots for select parameters from a house-level Bayesian model of *Mastomys natalensis* catch using only house trapping data from Sierra Leone and a house-level *Rattus rattus* predictor.** Site-, visit-, and house-level varying intercept parameters are not displayed for clarity. Plots show traces across all four independent Markov chains, with each chain represented by a different color. Note that the y-axis scale differs across parameters. In this model, the *R. rattus* effect is formulated as a house-level presence/absence variable (i.e., only houses where *R. rattus* was detected are assumed to be exposed to *R. rattus*).

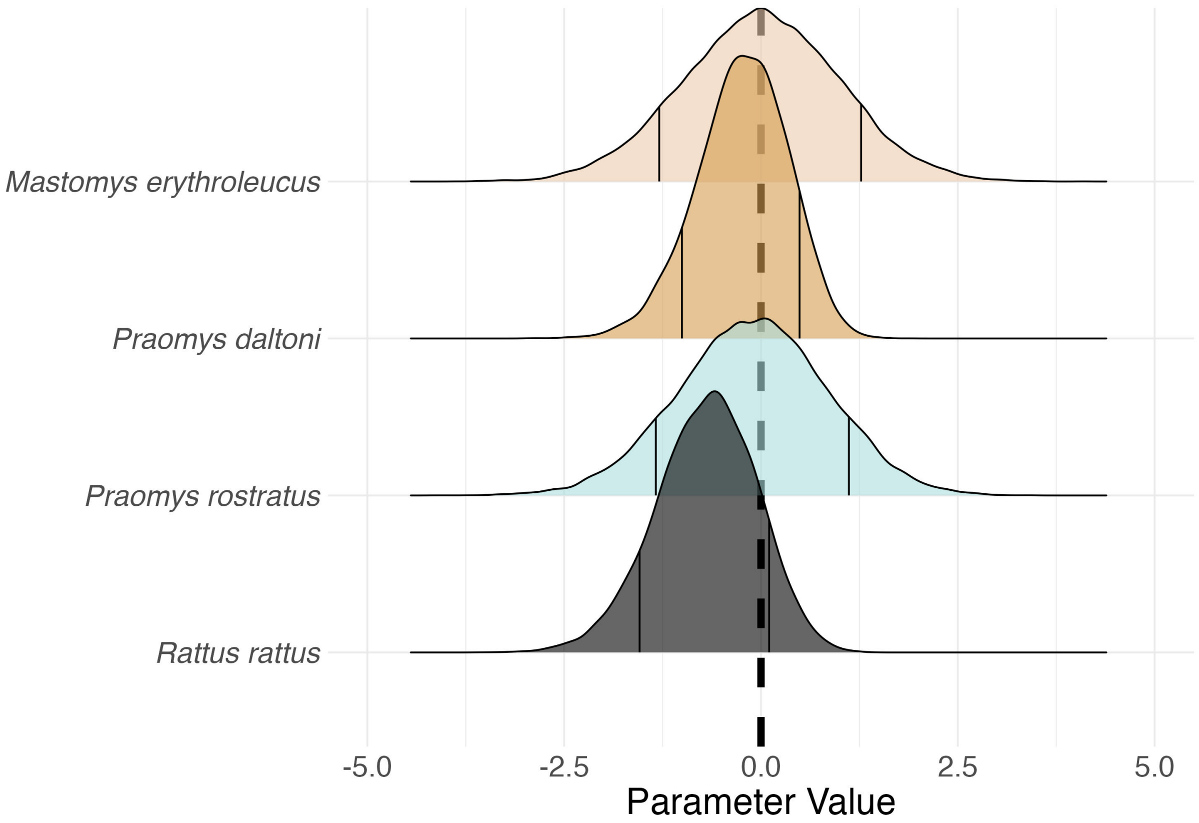

Figure S8: **Ridgeline plot showing estimated effects of the presence of four different rodent species on *Mastomys natalensis* catch using a house-level Bayesian model, house trapping data, and house-level rodent presence predictors.** To estimate these effects, we used a model identical to the house-level Bayesian model described in the main text, swapping out alternative rodent species for the *R. rattus* presence at house effect (which is also shown here). All rodents with 100 or more captures in our full dataset were tested, with the exception of *Mus mattheyi*, which was never captured in houses in Sierra Leone. The complete posterior distribution for each parameter is shown, and thin vertical lines within the distributions delineate the 80% percentile intervals (i.e., the 10th and 90th percentiles of each distribution).

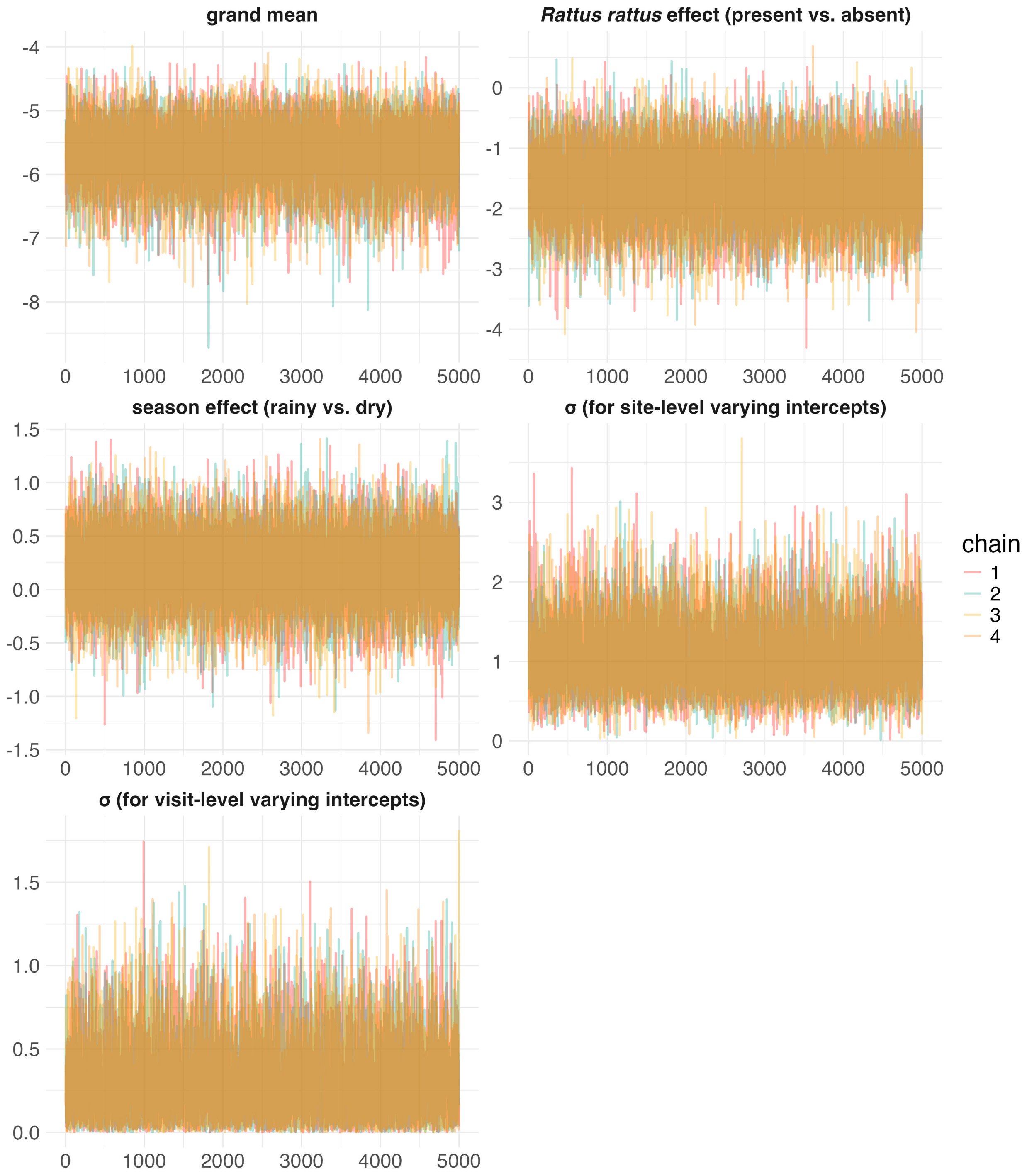

Figure S9: **Trace plots for select parameters from a visit-level Bayesian model of Lassa-positive *Mastomys natalensis* catch using only house trapping data.** Site- and visit-level varying intercept parameters are not displayed for clarity. Plots show traces across all four independent Markov chains, with each chain represented by a different color. Note that the y-axis scale differs across parameters.

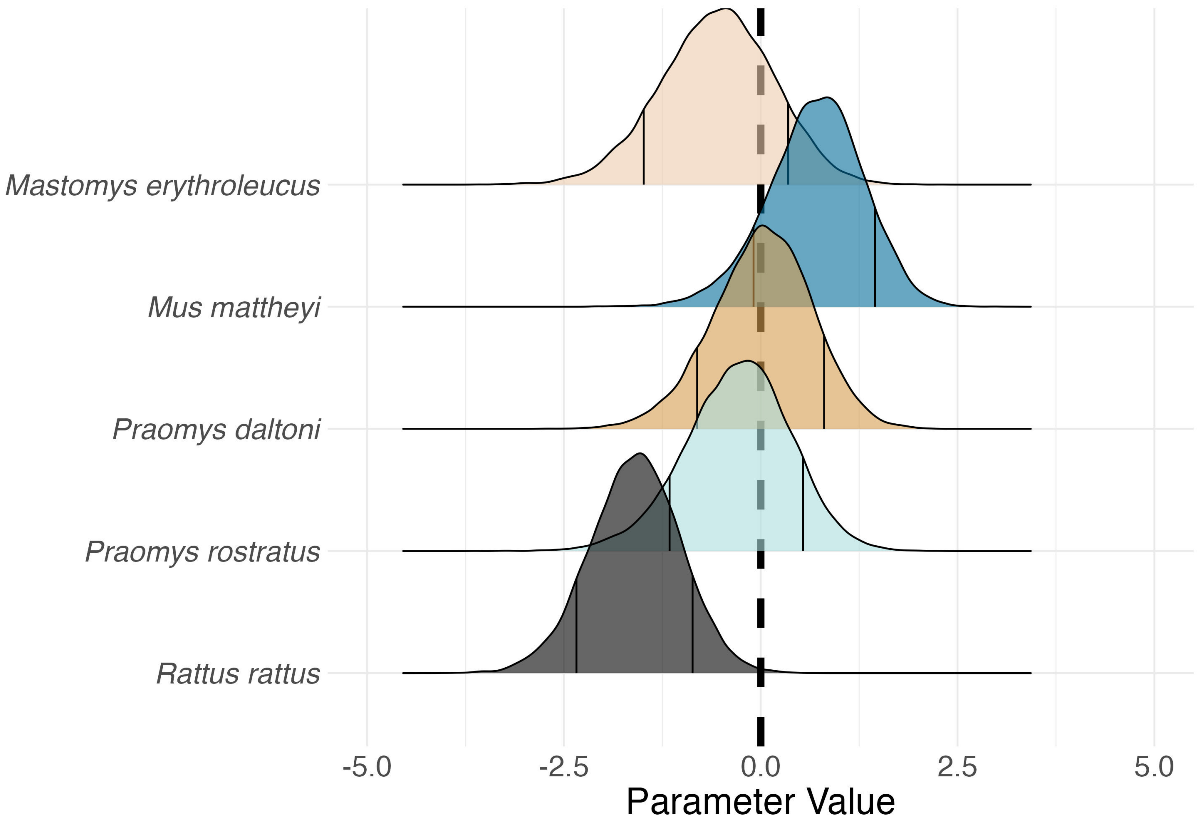

Figure S10: **Ridgeline plot showing estimated effects of the presence of five different rodent species on Lassa-positive *Mastomys natalensis* catch using a visit-level Bayesian model and house trapping data.** To estimate these effects, we used a model identical to the visit-level Bayesian model for Lassa-positive *M. natalensis* described in the main text, swapping out alternative rodent species for the *R. rattus* presence effect (which is also shown here). All rodents with 100 or more captures in our full dataset were tested. The complete posterior distribution for each parameter is shown, and thin vertical lines within the distributions delineate the 80% percentile intervals (i.e., the 10th and 90th percentiles of each distribution).

$$\text{Mna}_i \sim \text{Poisson}(\lambda_i)$$
[likelihood statement]

$$\log(\lambda_i) = \bar{\alpha} + \beta_{\text{Rra}} * \text{Rra}_i + \beta_{\text{Season}} * \text{Season}_i + \alpha_{\text{Site}[i]} + \alpha_{\text{Visit}[i]} + \log(\text{Tot}_i)$$
[linear model of λ]

$$\bar{\alpha} \sim \text{Normal}(-3.1, 1.1)$$
[prior for grand mean (i.e., intercept parameter)]

$$\beta_{\text{Rra}} \sim \text{Normal}(0, 1)$$
[prior for effect of *Rattus rattus* (present vs. absent at site)]

$$\beta_{\text{Season}} \sim \text{Normal}(0, 1)$$
[prior for effect of sampling season (rainy vs. dry)]

$$\alpha_j \sim \text{Normal}(0, \sigma_j)$$
[site-level varying intercepts]

$$\alpha_i \sim \text{Normal}(0, \sigma_i)$$
[visit-level varying intercepts]

$$\sigma_j \sim \text{Exponential}(1)$$
[hyperprior for standard deviation of site-level varying intercepts]

$$\sigma_i \sim \text{Exponential}(1)$$
[hyperprior for standard deviation of visit-level varying intercepts]

Figure S11: **A visit-level Bayesian model of *Mastomys natalensis* catch per trap.** For statistical modeling, data were organized into each of i total site visits occurring over j sites. The outcome data were the number of *M. natalensis* captured during a given site visit (Mna_i). $\bar{\alpha}$ represents the grand mean, or intercept parameter, and had an informative prior based on *M. natalensis* catch per trap values from previous studies (see Figure S12). Two binary main effects predictors were included: the presence/absence of *R. rattus* at a site (Rra_i) and the season of the site visit (Season_i). We also included varying intercepts (i.e., random effects) by site (α_j) and visit (α_i). Tot_i represents the trapping effort for a given site visit (i.e., total number of trap-nights) and was used as an offset term. A log link function was used to connect the Poisson rate parameter (λ) to the unbounded linear predictors.

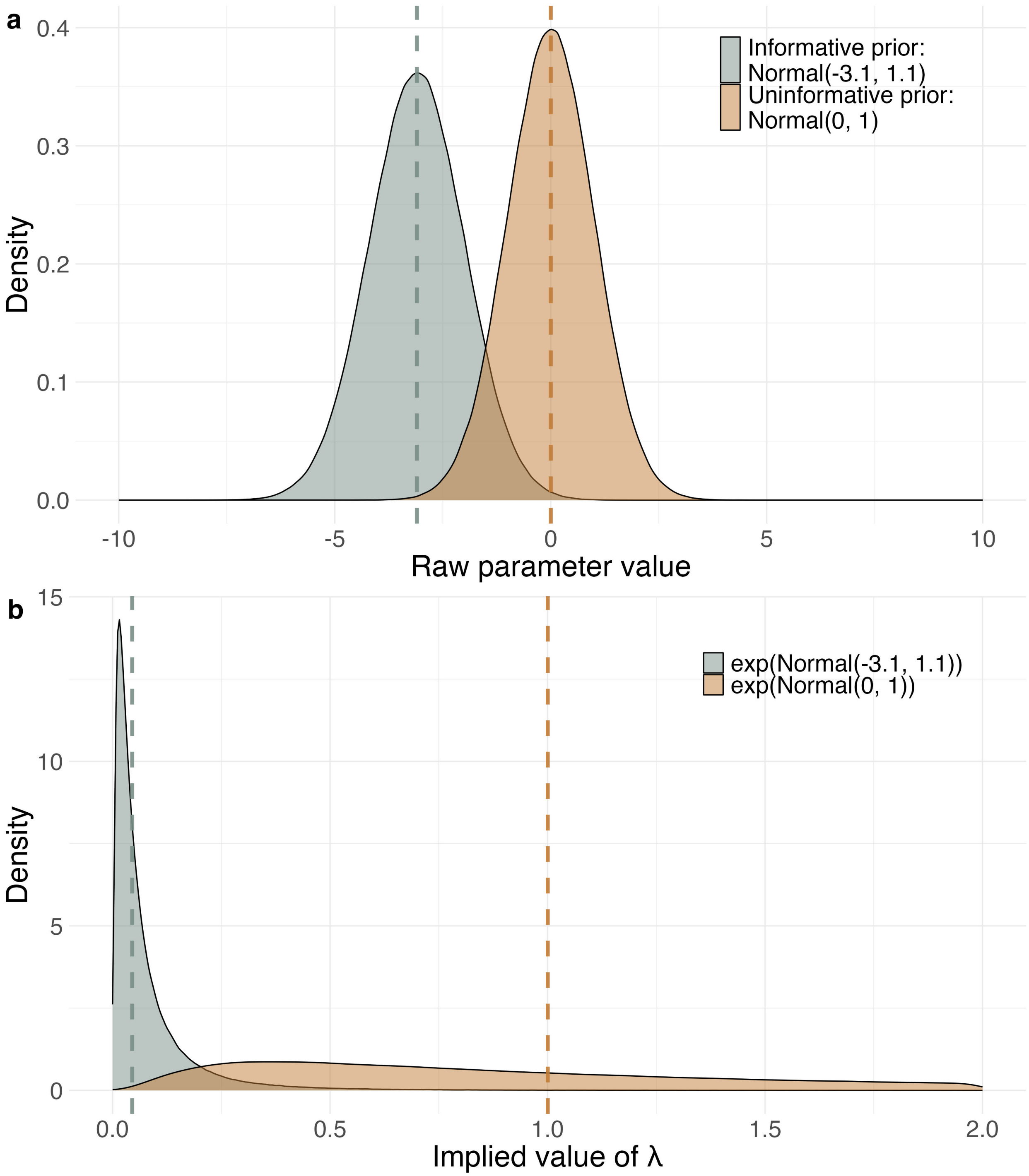

Figure S12: **Visualization of the informative prior used for the grand mean ($\bar{\alpha}$) parameter in Bayesian modeling.** Panel (a) compares the informative prior chosen based on previous rodent research (Normal(-3.1, 1.1)) to an uninformative prior (Normal(0, 1)). Panel (b) shows the Poisson rate (λ) distributions these two priors imply (i.e., following transformation through the model's inverse-link function). In both panels, vertical dashed lines represent distribution medians.

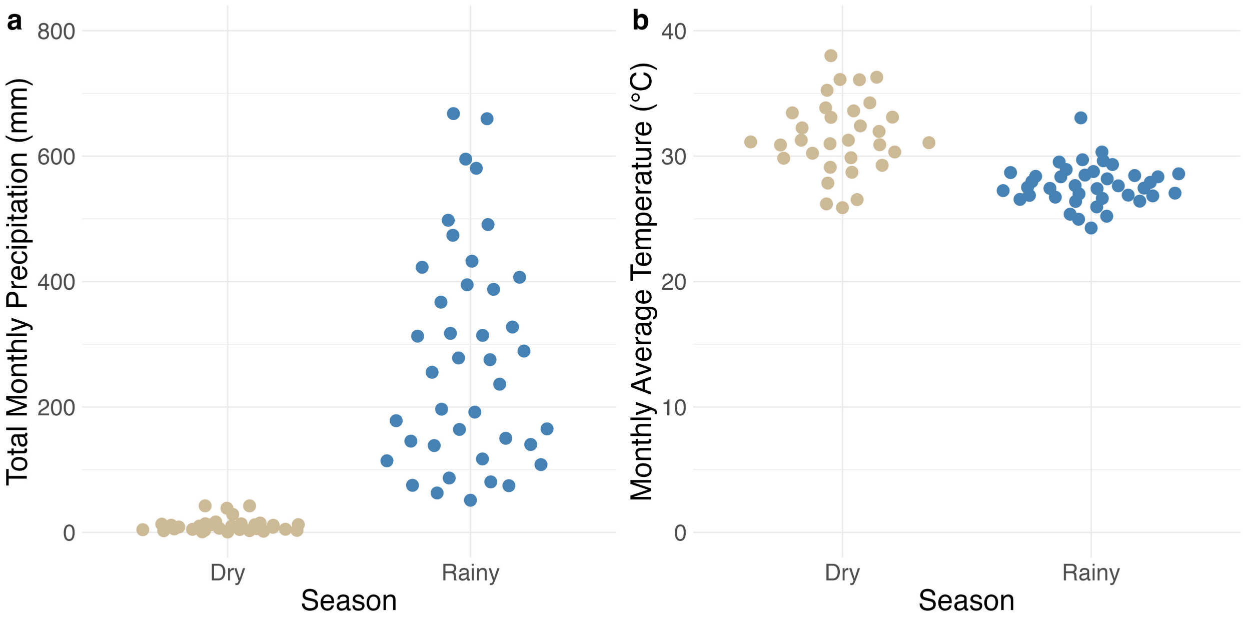

Figure S13: **Visualization of environmental data for all site visits (n = 72) from the visit-level *Mastomys natalensis* capture dataset.** Spatially-explicit data on the total precipitation for the month of each site visit (a) and the average monthly daytime temperature for the month of each site visit (b) were extracted from the CHIRPS and MODIS datasets, respectively. Two-sample *t*-tests showed that both precipitation ($t(70) = -8.70$, $P < 0.001$) and temperature ($t(70) = 7.08$, $P < 0.001$) differed significantly between dry and rainy season site visits using our definition of seasonality (dry season: December-April, rainy season: May-November).

Table S1: **GenBank accession numbers for Lassa virus sequence data supporting 51 PCR-positive *Mastomys natalensis* captured within homes.** For animals captured in Guinea, nucleoprotein (NP) sequence was generated, while for animals captured in Sierra Leone, complete sequences for the S and L genome segments were produced.

Country	Animal ID	GenBank NP accession	GenBank S segment accession	GenBank L segment accession
Guinea	129	KP339050		
Guinea	132	KP339051		
Guinea	148	KP339052		
Guinea	170	KP339054		
Guinea	263	KP339056		
Guinea	289	KP339057		
Guinea	302	KP339058		
Guinea	416	KP339071		
Guinea	417	KP339072		
Guinea	431	KP339074		
Guinea	444	KP339075		
Guinea	462	KP339076		
Guinea	464	KP339077		
Guinea	686	KP339081		
Guinea	717	KP339082		
Guinea	813	KP339083		
Guinea	817	KP339084		
Guinea	820	KP339085		
Guinea	846	KP339086		
Guinea	903	KP339087		
Guinea	909	KP339088		
Guinea	936	KP339089		
Guinea	939	KP339090		
Guinea	987	KP339093		
Guinea	1007	KP339095		
Guinea	1008	KP339096		
Guinea	1010	KP339097		
Guinea	1011	KP339098		
Guinea	1033	KP339099		
Guinea	1034	KP339100		
Guinea	1074	KP339102		
Guinea	1207	KP339104		
Guinea	1210	KP339105		
Guinea	1240	KP339106		
Guinea	1241	KP339107		
Guinea	1242	KP339108		
Guinea	1276	KP339110		
Guinea	1484	KP339112		
Guinea	1520	KP339113		
Guinea	1549	KP339114		
Guinea	1586	KP339115		
Guinea	1591	KP339116		
Guinea	1605	KP339117		
Guinea	1606	KP339118		
Guinea	1645	KP339119		
Sierra Leone	00106		OM735986	OM735987

Sierra Leone	00204	OM735980	OM735981
Sierra Leone	00214	OM735978	OM735979
Sierra Leone	00291	OM735972	OM735973
Sierra Leone	00292	OM735970	OM735971
Sierra Leone	00521	OM791222	OM791221
