# Supplementary material for: Reservoir displacement by an invasive rodent reduces Lassa virus zoonotic spillover risk
Source: Nat Commun. 2024 Apr 27;15:3589. doi: 10.1038/s41467-024-47991-1 (PMC11055883; doi:10.1038/s41467-024-47991-1)
Supplement: Supplementary file 3 — Reporting Summary [file 41467_2024_47991_MOESM3_ESM.pdf]

nature portfolio

Corresponding author(s): Evan Eskew

Last updated by author(s): Apr 9, 2024

Reporting Summary

Nature Portfolio wishes to improve the reproducibility of the work that we publish. This form provides structure for consistency and transparency in reporting. For further information on Nature Portfolio policies, see our Editorial Policies and the Editorial Policy Checklist.

Statistics

For all statistical analyses, confirm that the following items are present in the figure legend, table legend, main text, or Methods section.

n/a	Confirmed	
☐	☒	The exact sample size (*n*) for each experimental group/condition, given as a discrete number and unit of measurement
☐	☒	A statement on whether measurements were taken from distinct samples or whether the same sample was measured repeatedly
☐	☒	The statistical test(s) used AND whether they are one- or two-sided<br>*Only common tests should be described solely by name; describe more complex techniques in the Methods section.*
☐	☒	A description of all covariates tested
☐	☒	A description of any assumptions or corrections, such as tests of normality and adjustment for multiple comparisons
☐	☒	A full description of the statistical parameters including central tendency (e.g. means) or other basic estimates (e.g. regression coefficient) AND variation (e.g. standard deviation) or associated estimates of uncertainty (e.g. confidence intervals)
☐	☒	For null hypothesis testing, the test statistic (e.g. *F*, *t*, *r*) with confidence intervals, effect sizes, degrees of freedom and *P* value noted<br>*Give P values as exact values whenever suitable.*
☐	☒	For Bayesian analysis, information on the choice of priors and Markov chain Monte Carlo settings
☒	☐	For hierarchical and complex designs, identification of the appropriate level for tests and full reporting of outcomes
☒	☐	Estimates of effect sizes (e.g. Cohen's *d*, Pearson's *r*), indicating how they were calculated

Our web collection on statistics for biologists contains articles on many of the points above.

Software and code

Policy information about availability of computer code

Data collection	No specialized software was used for data collection in this study.
Data analysis	All data were analyzed in R (version 4.2.1) via the RStudio interface (version 2023.06.1+524). R packages used to analyze and visualize data include: 'assertthat' (version 0.2.1), 'cmdstanr' (version 0.5.3), 'cowplot' (version 1.1.2), 'data.table' (version 1.14.10), 'geosphere' (version 1.5-18), 'ggbeeswarm' (version 0.7.2), 'osmdata' (version 0.2.5), 'plyr' (version 1.8.9), 'readxl' (version 1.4.3), 'rethinking' (version 2.21), 'rnaturalearth' (version 1.0.1), 'rnaturalearthdata' (version 0.1.0), 'sf' (version 1.0-15), 'spOccupancy' (version 0.7.2), 'terra' (version 1.7-65), and 'tidyverse' (version 2.0.0). Bayesian models were fit with Stan (version 2.32.2) via the CmdStan interface (version 2.30.0). Code supporting the study is publicly available via the project GitHub (https://github.com/eveskew/rat_invasion) and Zenodo (https://doi.org/10.5281/zenodo.10946459) repositories.

For manuscripts utilizing custom algorithms or software that are central to the research but not yet described in published literature, software must be made available to editors and reviewers. We strongly encourage code deposition in a community repository (e.g. GitHub). See the Nature Portfolio guidelines for submitting code & software for further information.

Data

Policy information about availability of data

All manuscripts must include a data availability statement. This statement should provide the following information, where applicable:

- Accession codes, unique identifiers, or web links for publicly available datasets
- A description of any restrictions on data availability
- For clinical datasets or third party data, please ensure that the statement adheres to our policy

All data used to generate the analytical results in this manuscript are openly available via the project GitHub (https://github.com/eveskew/rat_invasion) and Zenodo (https://doi.org/10.5281/zenodo.10946459) repositories. GenBank accession codes for Lassa virus sequence data from infected Mastomys natalensis (also given in Table S1) are:
KP339050 [https://www.ncbi.nlm.nih.gov/nuccore/KP339050],
KP339051 [https://www.ncbi.nlm.nih.gov/nuccore/KP339051],
KP339052 [https://www.ncbi.nlm.nih.gov/nuccore/KP339052],
KP339054 [https://www.ncbi.nlm.nih.gov/nuccore/KP339054],
KP339056 [https://www.ncbi.nlm.nih.gov/nuccore/KP339056],
KP339057 [https://www.ncbi.nlm.nih.gov/nuccore/KP339057],
KP339058 [https://www.ncbi.nlm.nih.gov/nuccore/KP339058],
KP339071 [https://www.ncbi.nlm.nih.gov/nuccore/KP339071],
KP339072 [https://www.ncbi.nlm.nih.gov/nuccore/KP339072],
KP339074 [https://www.ncbi.nlm.nih.gov/nuccore/KP339074],
KP339075 [https://www.ncbi.nlm.nih.gov/nuccore/KP339075],
KP339076 [https://www.ncbi.nlm.nih.gov/nuccore/KP339076],
KP339077 [https://www.ncbi.nlm.nih.gov/nuccore/KP339077],
KP339081 [https://www.ncbi.nlm.nih.gov/nuccore/KP339081],
KP339082 [https://www.ncbi.nlm.nih.gov/nuccore/KP339082],
KP339083 [https://www.ncbi.nlm.nih.gov/nuccore/KP339083],
KP339084 [https://www.ncbi.nlm.nih.gov/nuccore/KP339084],
KP339085 [https://www.ncbi.nlm.nih.gov/nuccore/KP339085],
KP339086 [https://www.ncbi.nlm.nih.gov/nuccore/KP339086],
KP339087 [https://www.ncbi.nlm.nih.gov/nuccore/KP339087],
KP339088 [https://www.ncbi.nlm.nih.gov/nuccore/KP339088],
KP339089 [https://www.ncbi.nlm.nih.gov/nuccore/KP339089],
KP339090 [https://www.ncbi.nlm.nih.gov/nuccore/KP339090],
KP339093 [https://www.ncbi.nlm.nih.gov/nuccore/KP339093],
KP339095 [https://www.ncbi.nlm.nih.gov/nuccore/KP339095],
KP339096 [https://www.ncbi.nlm.nih.gov/nuccore/KP339096],
KP339097 [https://www.ncbi.nlm.nih.gov/nuccore/KP339097],
KP339098 [https://www.ncbi.nlm.nih.gov/nuccore/KP339098],
KP339099 [https://www.ncbi.nlm.nih.gov/nuccore/KP339099],
KP339100 [https://www.ncbi.nlm.nih.gov/nuccore/KP339100],
KP339102 [https://www.ncbi.nlm.nih.gov/nuccore/KP339102],
KP339104 [https://www.ncbi.nlm.nih.gov/nuccore/KP339104],
KP339105 [https://www.ncbi.nlm.nih.gov/nuccore/KP339105],
KP339106 [https://www.ncbi.nlm.nih.gov/nuccore/KP339106],
KP339107 [https://www.ncbi.nlm.nih.gov/nuccore/KP339107],
KP339108 [https://www.ncbi.nlm.nih.gov/nuccore/KP339108],
KP339110 [https://www.ncbi.nlm.nih.gov/nuccore/KP339110],
KP339112 [https://www.ncbi.nlm.nih.gov/nuccore/KP339112],
KP339113 [https://www.ncbi.nlm.nih.gov/nuccore/KP339113],
KP339114 [https://www.ncbi.nlm.nih.gov/nuccore/KP339114],
KP339115 [https://www.ncbi.nlm.nih.gov/nuccore/KP339115],
KP339116 [https://www.ncbi.nlm.nih.gov/nuccore/KP339116],
KP339117 [https://www.ncbi.nlm.nih.gov/nuccore/KP339117],
KP339118 [https://www.ncbi.nlm.nih.gov/nuccore/KP339118],
KP339119 [https://www.ncbi.nlm.nih.gov/nuccore/KP339119],
OM735986 [https://www.ncbi.nlm.nih.gov/nuccore/OM735986],
OM735987 [https://www.ncbi.nlm.nih.gov/nuccore/OM735987],
OM735980 [https://www.ncbi.nlm.nih.gov/nuccore/OM735980],
OM735981 [https://www.ncbi.nlm.nih.gov/nuccore/OM735981],
OM735978 [https://www.ncbi.nlm.nih.gov/nuccore/OM735978],
OM735979 [https://www.ncbi.nlm.nih.gov/nuccore/OM735979],
OM735972 [https://www.ncbi.nlm.nih.gov/nuccore/OM735972],
OM735973 [https://www.ncbi.nlm.nih.gov/nuccore/OM735973],
OM735970 [https://www.ncbi.nlm.nih.gov/nuccore/OM735970],
OM735971 [https://www.ncbi.nlm.nih.gov/nuccore/OM735971],
OM791222 [https://www.ncbi.nlm.nih.gov/nuccore/OM791222],
OM791221 [https://www.ncbi.nlm.nih.gov/nuccore/OM791221].
CHIRPS precipitation (https://www.chc.ucsb.edu/data/chirps) and MODIS land surface temperature (https://lpdaac.usgs.gov/products/mod11a1v061/) data used to validate our seasonality variable are freely available online and are also included in our project's Zenodo repository (https://doi.org/10.5281/zenodo.10946459).
Source data are provided with this paper.

Research involving human participants, their data, or biological material

Policy information about studies with human participants or human data. See also policy information about sex, gender (identity/presentation), and sexual orientation and race, ethnicity and racism.

Reporting on sex and gender	This study did not involve data from human participants.
Reporting on race, ethnicity, or other socially relevant groupings	This study did not involve data from human participants.
Population characteristics	This study did not involve data from human participants.
Recruitment	This study did not involve data from human participants.
Ethics oversight	This study did not involve data from human participants.

Note that full information on the approval of the study protocol must also be provided in the manuscript.

Field-specific reporting

Please select the one below that is the best fit for your research. If you are not sure, read the appropriate sections before making your selection.

☐ Life sciences ☐ Behavioural & social sciences ☒ Ecological, evolutionary & environmental sciences

For a reference copy of the document with all sections, see nature.com/documents/nr-reporting-summary-flat.pdf

Ecological, evolutionary & environmental sciences study design

All studies must disclose on these points even when the disclosure is negative.

Study description	This study leverages rodent trapping data from 17 village sites in Sierra Leone and 11 village sites in Guinea to examine catch per trap of Mastomys natalensis, the primary reservoir host of Lassa virus, in relation to the presence of the invasive rodent Rattus rattus. A complementary analysis treats the number of Lassa virus positive Mastomys natalensis as an index of viral spillover risk to humans. As this was not an experimental study, there were no treatments applied. Instead, analyses focus on Mastomys natalensis catch per trap at sites where Rattus rattus was detected (n = 18 sites) compared to sites where Rattus rattus was never detected (n = 10 sites).
Research sample	Broadly, the relevant research sample is all wild rodents living in and around the 28 village sites in Sierra Leone and Guinea. However, for this particular study we focus on two key rodent species, Mastomys natalensis and Rattus rattus, given existing scientific literature that hypothesizes antagonistic interactions between these two species. For both of these two focal species, both sexes and animals of all ages were considered in our analyses. Rodent trapping data used in the analyses are available on both the project GitHub (https://github.com/eveskew/rat_invasion) and Zenodo (https://doi.org/10.5281/zenodo.10946459) repositories.
Sampling strategy	Animals and biological samples were collected by deploying live animal traps in three distinct areas within each village: within houses, along trap-lines within the village perimeter, and along trap-lines outside of the village perimeter. Given repeated sampling within some sites (see "Timing and spatial scale" section below), site-level trapping effort ranged from 211 to 6,303 trap-nights. Sampling effort at each site was informed by prior rodent trapping work but also constrained by the logistical challenges of ecological fieldwork in our study region. Sample sizes were not predetermined for this particular research study as it relied on existing rodent trapping data. Instead, we leveraged all Mastomys natalensis (n = 950) and Rattus rattus captures (n = 151) available to us. Sampling effort (i.e., trap-nights) and sample size (i.e., rodent captures) are explicitly incorporated into our Bayesian modeling framework and posterior estimates from our models naturally reflect the effects of sample size (i.e., results driven by sites with less sampling would have wider posterior distributions, indicating greater uncertainty).
Data collection	During trapping periods, traps were checked every day in the early morning. Animal counts were recorded. All further data collection in the field was performed using methods analogous to BSL-3 protocols. Species-level identification was initially assigned based on morphological characteristics and later confirmed via cytochrome b sequencing. Sierra Leone team data were recorded on paper data collection tools (including location, animal morphometric, and environmental data). After completion of each sampling event, physical data were converted to electronic data storage for later analyses. Paper copies were subsequently scanned and stored electronically as primary data sources for long-term storage. Each data set was reviewed by at least two independent Sierra Leone-based project staff workers to ensure data quality and accuracy. A final data review and quality assurance review was completed by UC Davis project staff. For Guinea data, Elisabeth Fichet-Calvet (EFC) recorded the data with a pencil on paper, using trapping, necropsy, and biopsy data sheets. The sheets were disinfected with ethanol each day after necropsy sessions. EFC transferred the data from paper data sheets into Excel files, entering the variables in columns (vertically). Once the data had been entered electronically, a quality check was carried out by reading the data horizontally to remove errors from the database. For Guinea data, the morphological identification of the rodents at the species level was carried out by EFC in the field, while the molecular confirmation of species identity was performed by Emilie Lecompte in Marburg, Germany. In Sierra Leone, oral/urogenital/rectal swabs and blood samples collected from captured animals were all tested using the real-time PCR primers and protocol of Nikisins et al. 2015. If any specimen from an individual rodent returned positive results, that animal was deemed Lassa virus positive. In Guinea, rodent blood was tested for Lassa virus using conventional RT-PCR according to the protocol of Vieth et al. 2007. Lassa PCR positive

animals from both Sierra Leone and Guinea were further confirmed by viral sequencing, as described in the manuscript.

Timing and spatial scale

In Sierra Leone, sites were visited between 1 and 5 times between July 2019 and February 2021. Site visits occurred in all months of the year except for April-June. In Guinea, sites were visited between 1 and 6 times between October 2002 and February 2005. Generally, site visits occurred in January, May, or October. For the project generally, site visits were scheduled to adequately capture seasonal variation across the study region while being constrained by the logistical challenges of field sampling. Site visitation effort varied based on rodent trapping success in prior sampling periods. Data were collected and recorded at the level of individual traps but were analyzed at aggregated scales, either at the visit- or house-levels.

Data exclusions

Data were not excluded from the study except to facilitate the analysis of specific data subsets (i.e., models using trapping data only from houses), as reported in the manuscript. Analysis of the full trapping dataset is included in the Supplementary Information.

Reproducibility

As an observational ecological study, there was no attempt to replicate the study as a whole. However, we did attempt to bolster the study's conclusions by reproducing the general analysis strategy (examining the impact of Rattus rattus presence on Mastomys natalensis catch) across scales using different analytical methods. More specifically, we conducted analyses using all traps at the visit-level (using a site-level Rattus rattus presence predictor), using only house traps at the visit-level (using a site-level Rattus rattus presence predictor), and using only house traps at the house-level (using both site-level and house-level Rattus rattus presence predictors). In addition, where data specificity allowed, we conducted an occupancy analysis on trapping data obtained from repeated sampling from within the same human habitations. Finally, our study aims for reproducibility by the community at large by making our analysis code and data publicly available via GitHub (https://github.com/eveskew/rat_invasion) and Zenodo (https://doi.org/10.5281/zenodo.10946459).

Randomization

Randomization is not relevant to this observational ecological study where the primary predictor of interest (Rattus rattus presence) had to be determined through field sampling.

Blinding

Blinding is not relevant to our study design. Those collecting data in the field could not be blinded to the presence of the animal species which they were tasked with identifying.

Did the study involve field work? ☒ Yes ☐ No

Field work, collection and transport

Field conditions

Field conditions varied as field work in Sierra Leone took place from July 2019 to February 2021 while field work in Guinea took place from October 2002 to February 2005. For analyses, all site visits (n = 72) were categorized into rainy (n = 40) or dry season (n = 32) visits. Data on the total precipitation and average daytime temperature for the month of each site visit are shown in Figure S13. Briefly, total precipitation for the month of each site visit averaged 161.04 mm (range: 0.57-667.81 mm) while daytime temperature for the month of each site visit averaged 29.43 Celsius (range: 24.27-38.01 Celsius).

Location

Field work was conducted across 17 sites in Sierra Leone and 11 sites in Guinea. The locations of the 28 study sites, given as longitude/latitude (also available via GitHub: https://github.com/eveskew/rat_invasion/blob/main/data/clean/combined/site_level_data.csv), are:

Sierra Leone:
Badala, -11.54076407 9.31972941
Bafodia, -11.7330408 9.683164617
Barlie, -11.58421744 8.019789811
Benduma, -11.52150336 8.025988093
Gbainkfay, -12.25657928 9.298792229
Gbenikoro, -11.47186785 9.622999692
Guala, -11.23562166 8.124279287
Kapethe, -11.98241063 8.986206142
Largo, -11.10338553 8.051547331
Makump, -12.03982493 8.805581045
Makuna, -11.98429841 8.748832258
Mokorie, -12.05804871 8.207852394
Naiawama, -11.04104694 7.869442223
Njaguima, -11.64293135 8.424344885
Petema, -11.62825816 7.747475129
Talama, -11.12266698 8.195003613
Yekeyor, -10.87848581 8.576877643

Guinea:
Bamba, -13.885 10.00055556
Bantou, -10.57866574 10.05451515
Gagal, -12.2975 11.08805556
Gania, -12.53963981 10.06655741
Gayebombo, -13.59083333 10.13231481
Gbetaya, -11.04 9.840833333
Kaali, -12.70597222 9.930416667
Khoneya, -12.67583333 10.14861111
Sangassou, -9.474166667 8.613611111

	Tanganya, -10.97318918 10.0007123<br>Yafraya, -13.67888889 10.01194444
Access & import/export	Animal work in Sierra Leone was conducted in collaboration with the Sierra Leone Ministry of Health and Sanitation and the Ministry of Agriculture and Forestry under permit number CONF/LSD/02/17. Field work in Guinea was authorized by the Ministry of Public Health under permit number 2003/PFHG/05/GUI.
Disturbance	Rodent trapping caused minimal disturbance to the field environment.

Reporting for specific materials, systems and methods

We require information from authors about some types of materials, experimental systems and methods used in many studies. Here, indicate whether each material, system or method listed is relevant to your study. If you are not sure if a list item applies to your research, read the appropriate section before selecting a response.

Materials & experimental systems

n/a	Involved in the study
☒	☐ Antibodies
☒	☐ Eukaryotic cell lines
☒	☐ Palaeontology and archaeology
☐	☒ Animals and other organisms
☒	☐ Clinical data
☒	☐ Dual use research of concern
☒	☐ Plants

Methods

n/a	Involved in the study
☒	☐ ChIP-seq
☒	☐ Flow cytometry
☒	☐ MRI-based neuroimaging

Animals and other research organisms

Policy information about studies involving animals; ARRIVE guidelines recommended for reporting animal research, and Sex and Gender in Research

Laboratory animals	This study did not involve laboratory animals.
Wild animals	Animals were captured at village sites using live animal traps. Although multiple species were captured during field sampling efforts, this study focuses on data regarding Mastomys natalensis and Rattus rattus. Captured animals were processed in the field according to standard procedures for BSL-3-level work in such settings. The majority of animals were euthanized with isoflurane or halothane because: 1) this allowed collection of tissues including lung, liver, and spleen, and 2) these animals represent a zoonotic disease threat to the local human community.
Reporting on sex	Sex-specific or sex-based analyses were not performed in this work as the primary outcome of interest was the abundance of Mastomys natalensis, regardless of sex. This analytical decision is justified by the fact that both sexes of Mastomys natalensis may host Lassa virus and therefore represent a zoonotic spillover risk to humans.
Field-collected samples	This study did not involve laboratory work with field-collected animals. All relevant data and samples were collected from captured animals in the field, as described above and in the manuscript.
Ethics oversight	Animal work in Sierra Leone was approved by the University of California, Davis Institutional Animal Care and Use Committee (IACUC; protocol number 22696) and was conducted in collaboration with the Sierra Leone Ministry of Health and Sanitation and the Ministry of Agriculture and Forestry under permit number CONF/LSD/02/17. Field work in Guinea was authorized by the Ministry of Public Health under permit number 2003/PFHG/05/GUI.

Note that full information on the approval of the study protocol must also be provided in the manuscript.
